# *Karenia brevis* allelopathy compromises the lipidome, membrane integrity, and photosynthesis of competitors

**DOI:** 10.1038/s41598-018-27845-9

**Published:** 2018-06-22

**Authors:** Remington X. Poulin, Scott Hogan, Kelsey L. Poulson-Ellestad, Emily Brown, Facundo M. Fernández, Julia Kubanek

**Affiliations:** 1 School of Chemistry and Biochemistry, Georgia Institute of Technology, 901 Atlantic Dr, Atlanta, GA 30332 USA; 2 School of Biological Sciences, Georgia Institute of Technology, 950 Atlantic Dr, Atlanta, GA 30332 USA; 3 Department of Biological, Chemical, and Physical Sciences, Roosevelt University, 430S Michigan Avenue, Chicago, IL 60605 USA; 4 Aquatic Chemical Ecology Center, Georgia Institute of Technology, Atlanta, GA 30332 USA; 5 Parker H. Petit Institute of Bioengineering and Bioscience, Georgia Institute of Technology, Atlanta, GA 30332 USA

## Abstract

The formation, propagation, and maintenance of harmful algal blooms are of interest due to their negative effects on marine life and human health. Some bloom-forming algae utilize allelopathy, the release of compounds that inhibit competitors, to exclude other species dependent on a common pool of limiting resources. Allelopathy is hypothesized to affect bloom dynamics and is well established in the red tide dinoflagellate *Karenia brevis*. *K*. *brevis* typically suppresses competitor growth rather than being acutely toxic to other algae. When we investigated the effects of allelopathy on two competitors, *Asterionellopsis glacialis* and *Thalassiosira pseudonana*, using nuclear magnetic resonance (NMR) spectroscopy and mass spectrometry (MS)-based metabolomics, we found that the lipidomes of both species were significantly altered. However, *A*. *glacialis* maintained a more robust metabolism in response to *K*. *brevis* allelopathy whereas *T*. *pseudonana* exhibited significant alterations in lipid synthesis, cell membrane integrity, and photosynthesis. Membrane-associated lipids were significantly suppressed for *T*. *pseudonana* exposed to allelopathy such that membranes of living cells became permeable. *K*. *brevis* allelopathy appears to target lipid biosynthesis affecting multiple physiological pathways suggesting that exuded compounds have the ability to significantly alter competitor physiology, giving *K*. *brevis* an edge over sensitive species.

## Introduction

Harmful algal blooms, dense congregations of marine phytoplankton that often produce noxious compounds, can be toxic to human and marine life and are becoming increasingly frequent^1^. In addition to killing fish, seabirds, and marine mammals, potent algal toxins exuded by bloom-forming phytoplankton accumulate in filter feeders and predators, eventually entering the human food web through contaminated seafood such as clams, oysters, and fish^2–6^. Toxins produced by some algae also persist in ambient waters, affecting human health through exposure to toxin-containing aerosols and cellular debris, leading to respiratory distress^3,7–9^.

Allelopathy, the release of compounds into the surrounding water that negatively affect competitors, is an important manifestation of competition that influences aquatic community structure^10^. Allelopathy can significantly alter species composition^11,12^ and species succession^13,14^ through lethal interactions^14,15^, as well as sub-lethal outcomes such as reduced growth^16,17^, induction of cyst formation^18^, and suppression of swimming behavior^19,20^. Little is known of the compounds responsible for allelopathy in the marine plankton or the molecular targets of allelopathy despite significant research^21^. Typically, toxins produced by these phytoplankton have not been shown to be allelopathic, with the exception of karlotoxins produced by the dinoflagellate *Karlodinium veneficum*^22^. A more thorough understanding of the modes of action of allelopathy and of the compounds responsible could enhance our understanding of the roles and ecosystem-wide impacts of allelopathy in algal blooms.

*Karenia brevis*, a dinoflagellate that blooms in the Gulf of Mexico and Southeastern Atlantic Ocean, produces a suite of neurotoxins called brevetoxins that cause neurotoxic shellfish poisoning in humans and marine life^23^. Despite their toxicity, brevetoxins are not responsible for observed allelopathic effects of *K*. *brevis* on competitors^24–26^. Instead, allelopathic compounds are yet uncharacterized fatty acid-derived lipids and aromatic molecules whose lack of stability has prevented complete characterization^26^. Potency of *K*. *brevis* allelopathy varies among blooms, strains, and cultured batches and its effects are selective towards certain competitor species in the early stages of growth^24,27,28^. Reduced growth, membrane integrity, and photosynthetic efficiency have previously been reported for allelopathy-affected competitors; however, the molecular explanation for these physiological responses is yet poorly understood^27,29^. *K*. *brevis* is a particularly useful model for addressing the metabolic responses of competitors to allelopathy as the effects of *K*. *brevis* exposure are sub-lethal^24,25,28,29^. Metabolomics can provide a snapshot of alterations to the collection of small molecule metabolites in an organism during allelopathic stress, providing broad, system-level insights into possible mechanisms of action and causative agents of physiological responses.

Using proteomics as well as analysis of the polar metabolomes of two competing phytoplankton, *Asterionellopsis glacialis* and *Thalassiosira pseudonana*, we previously showed that energy metabolism, osmoregulation, photosynthesis, and fatty acid synthesis were all disrupted due to *K*. *brevis* allelopathy^29^. Notably, various aspects of lipid metabolism appeared to be disrupted, with enhanced concentrations of enzymes associated with lipid anabolism, such as sulpholipid synthase and UDP-sulfoquinovose synthase, and decreased concentrations of certain lipids, such as terpene glycosides, when *T*. *pseudonana* was exposed to allelopathy^29^. This previous study, while providing significant insights into the molecular targets and metabolic pathways affected by allelopathy, was biased towards polar metabolites and therefore unable to fully describe the effect of allelopathy on competitor phytoplankton. The disruption of lipid metabolic enzymes and pathways has been shown to drive many human diseases and disorders, including diabetes, many forms of cancer, neurodegenerative disease, and infectious diseases^30,31^. Additionally, changes in lipid concentrations and the use of various lipids as signals is common in plants^32,33^, as well as in phytoplankton during stress^34,35^.

The previously observed complex response of lipid metabolism to allelopathy^29^, coupled with the importance of the lipidome in regulating stress responses in many systems, led us to investigate the impacts of *K*. *brevis* allelopathy on the lipidome of competitors. Using nuclear magnetic resonance (NMR) spectroscopy and mass spectrometry (MS)-based metabolomics we identified the major metabolic effects of *K*. *brevis* allelopathy on the lipidomes (i.e., nonpolar metabolites) of two competitors, *A. glacialis*, which co-occurs with *K*. *brevis* during blooms and *T. pseudonana*, which originates from non-bloom areas, in order to achieve a more complete understanding of the physiological responses and mode of action of allelopathy. Insights from these new analyses include not only that the magnitude of the allelopathic effect is much more pronounced on the lipidome than on polar metabolites, and also that cell membrane stability, lipid metabolism, and thylakoid-associated lipid concentrations are dramatically impacted by allelopathy.

## Results

### Allelopathy affects the lipidomes of competing phytoplankton

**Figure 1:**
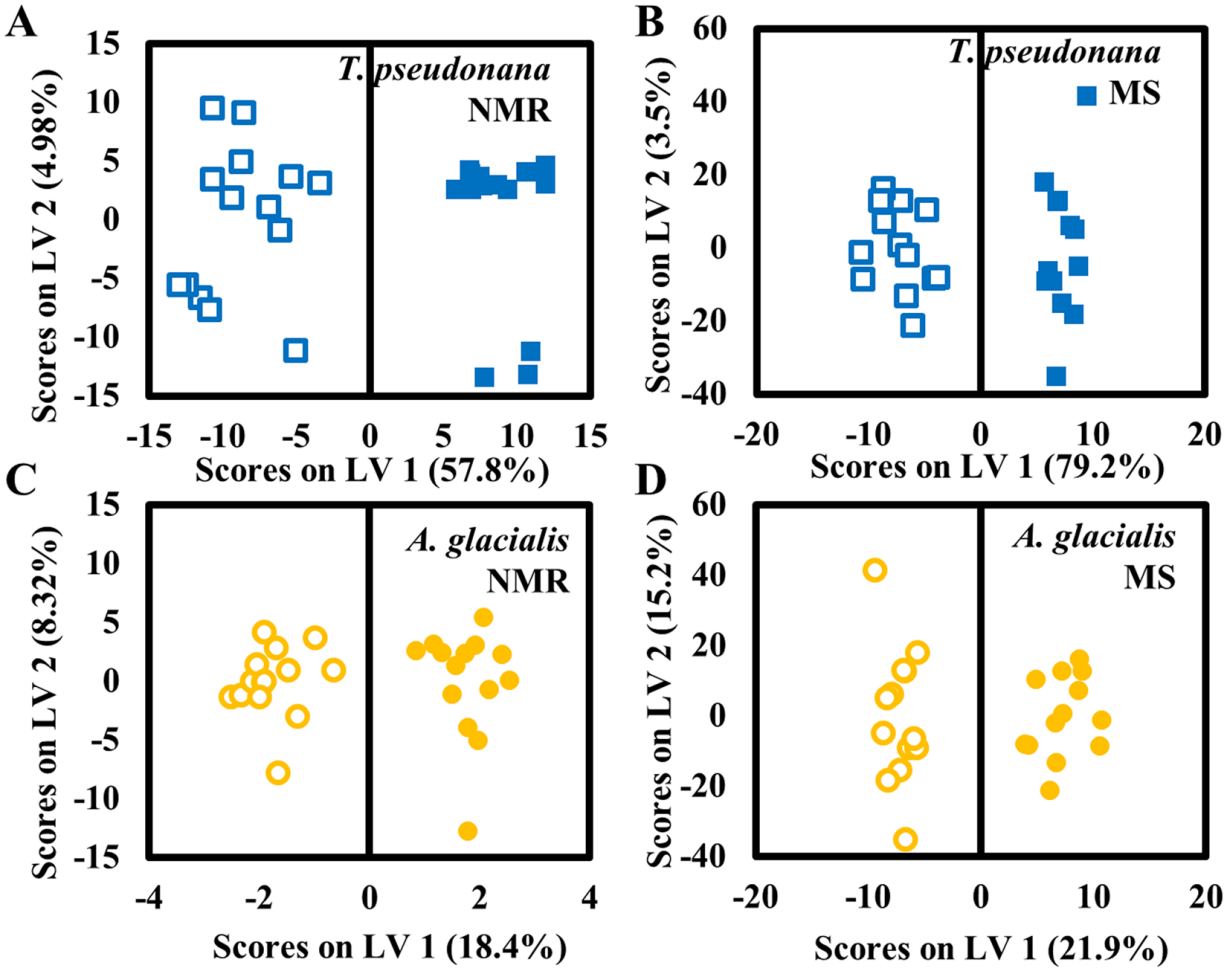
oPLS-DA models reveal that lipidomes of *Thalassiosira pseudonana* and *Asterionellopsis glacialis* are disrupted by *Karenia brevis* allelopathy. Filled symbols represent lipidomes of algae exposed to *K*. *brevis* through molecule-permeable but cell impermeable membranes, empty symbols represent lipidomes from unexposed algae (controls). oPLS-DA model generated from (**A**) ^1^H NMR spectral data and (**B**) from UHPLC/MS metabolic features from lipidomes of *T*. *pseudonana* (blue squares; variance captured along each latent variable is stated in parentheses). oPLS-DA model generated from (**C**) ^1^H NMR spectral data and (**D**) from UHPLC/MS metabolic features from lipidomes of *A*. *glacialis* (yellow circles).

In the current study, *K*. *brevis* allelopathy significantly affected the lipidomes of both competitor species *T*. *pseudonana* and *A*. *glacialis* (Fig. 1), even more dramatically than was previously observed for polar metabolites^29^. When we employed a multi-platform metabolomics approach combining NMR spectroscopy and MS, algae exposed to *K*. *brevis* allelopathy were distinguished from algae grown alone based on chemical dissimilarities in their lipidomes (Fig. 1). Orthogonal partial least squares discriminant analysis (oPLS-DA) identified a subset of spectral features corresponding to putatively identified metabolites that differentiated algae exposed to *K*. *brevis* allelopathy from controls. The concentration of any individual molecule alone could not comprehensively describe the differences in plankton lipidomes, but when combined as part of a discriminating panel they accurately differentiated the treatment effects of exposure to *K*. *brevis* allelopathy.

Principal component analysis (PCA) models were also generated; however, the discriminating power of most of these models was insufficient to identify algae based on their exposure to *K*. *brevis* allelopathy (Fig. S1). Despite this lack of discriminatory power, PCA of *T*. *pseudonana* metabolome profiles obtained via ultra-high performance liquid chromatography mass spectrometry (UHPLC/MS) accurately classified algae as having been exposed or unexposed to *K*. *brevis* (Fig. S1B). However, the rest of these PCA scores plots thus suggested the need for supervised multivariate classification tools to describe differences in the lipidomes, as well as to determine which variables or features were most critical in distinguishing between exposed and unexposed algae. Because MS is more sensitive than NMR spectroscopy and therefore allowed detection of differences in lower abundance metabolite concentrations, the ability of the MS-based PCA model to successfully differentiate between *T*. *pseudonana* exposed or not exposed to *K*. *brevis* was not surprising, and consistent with previous findings^29^.

### Metabolic responses to allelopathy are conserved among competitors, despite differential sensitivities

**Figure 2:**
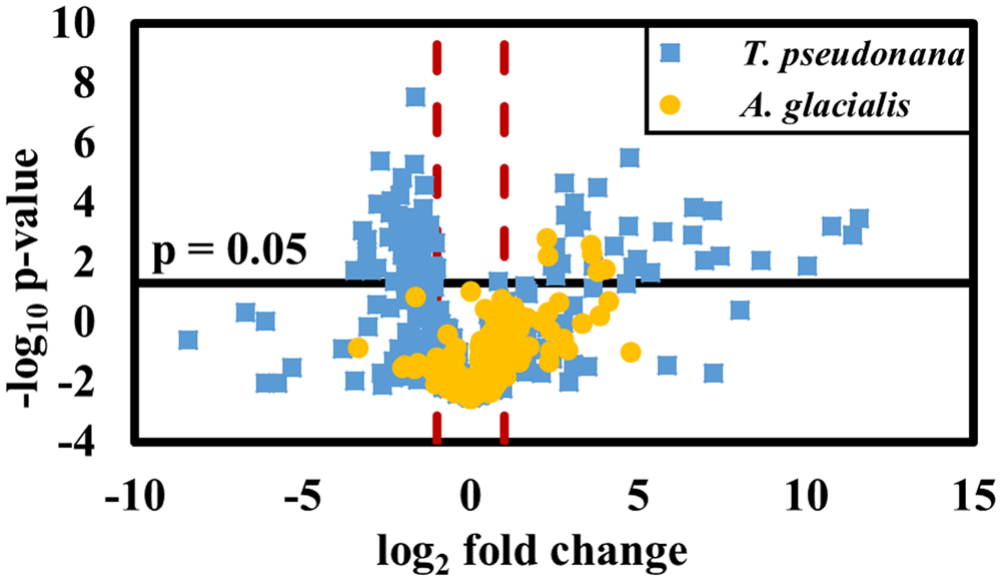
Volcano plot summarizes the differences in the lipdome of *T*. *pseudonana* (blue squares) and *A*. *glacialis* (yellow circles) when exposed vs. not exposed to *K*. *brevis* allelopathy. The relative abundances of 80 metabolites were significantly different (p < 0.05 after Bonferroni correction, see SI Materials and Methods) in *T*. *pseudonana* upon exposure to *K*. *brevis* allelopathy. Red lines indicate log_2_ fold difference of ±1. Six metabolites with concentrations that were significantly different in *A*. *glacialis* when exposed to *K*. *brevis* were also significantly different in concentration when *T*. *pseudonana* was exposed to *K*. *brevis*.

MS-based metabolomics analysis of lipidomes led to identification of 80 lipid metabolites whose concentrations differed significantly in *T*. *pseudonana* depending on exposure to *K*. *brevis* allelopathy (Table S1). Of these metabolites, 33 were significantly more abundant whereas 47 were significantly less abundant when *T*. *pseudonana* experienced *K*. *brevis* allelopathy (Fig. 2), with many of the metabolites exhibiting considerable fold differences due to *K*. *brevis* exposure (Table 1). These 80 metabolites represent nine major lipid classes, of which members of five (phosphatidylcholines [PCs], sulfoquinovosyldiacylglycerides [SQDGs], monogalactosyldiacylglycerides [MGDGs], digalactosyldiacylglycerides [DGDGs], and phosphatidylglycerols [PGs]) were generally less abundant when *T*. *pseudonana* was subjected to *K*. *brevis* allelopathy, whereas members of four classes (non-SQDG sulfonated lipids [SULF], free fatty acids [FFAs], primary fatty acid amides [PFAAs], and phosphatidylethanolamines [PEs]) were generally more abundant due to allelopathy (Table 1). In contrast, for the other competitor, *A*. *glacialis*, concentrations of only six metabolites were significantly affected by allelopathy, reinforcing that *A*. *glacialis* maintains a more robust metabolism in response to *K*. *brevis* allelopathy (Fig. 2, Tables S1 and S2). All six *A*. *glacialis* lipid metabolites whose concentrations varied with exposure to allelopathy were also modulated in *T*. *pseudonana*, all experiencing enhanced concentrations in both competitor species due to *K*. *brevis* exposure.

**Table 1:** Lipid classes identified by MS-based oPLS-DA model as having significantly different concentrations in *T*. *pseudonana* based upon exposure to *K*. *brevis* allelopathy.

Lipid Class	Adduct Detected	#Chemical Species	Average Fold Change
PC	[M + HCOO]^−^	15	−5.8
SQDG	[M − H]^−^	14	−3.3
DGDG	[M + HCOO]^−^	3	−3.3
PG	[M − H]^−^	3	−2.9
MGDG	[M + HCOO]^−^	12	−2.8
PE	[M + HCOO]^−^	1	6.5
PFAA	[M − H]^−^	5	25
FFA	[M − H]^−^	3	>25*
SULF	[M − H]^−^	14	>25*

Common adducts used to identify class of compound, the number of compounds in each class of lipids, and the average fold change is included for each class. The average fold change is an average of the individual fold changes each lipid identified in a class. Classes of lipids identified include: phosphatidylcholines (PCs), sulfoquinovosyldiacylglycerides (SQDGs), digalactosyldiacylglycerides (DGDGs), phosphatidylglycerols (PGs), monogalatoyldiacylglycerides (MGDGs), phosphatidylethanolamines (PEs), primary fatty acid amides (PFAAs), free fatty acids (FFAs), and non-SQDG sulfonated lipids (SULF).

*Fold change value uncertain due to extremely low concentration of metabolites in control samples.

In addition to observing mass spectral features that represent SQDGs, MGDGs, DGDGs, PCs, and PGs as major lipid classes affected by allelopathy (Table 1), five additional metabolites whose concentrations were suppressed in *T*. *pseudonana* by *K*. *brevis* allelopathy were revealed only by NMR spectroscopic analysis. They were identified as aconitic acid, malonic acid, methyl guanidine, *N*-acetyl cysteine, and *N*-acetyl glutamine.

### Cell membrane integrity is weakened by *K*. *brevis* allelopathy

**Figure 3:**
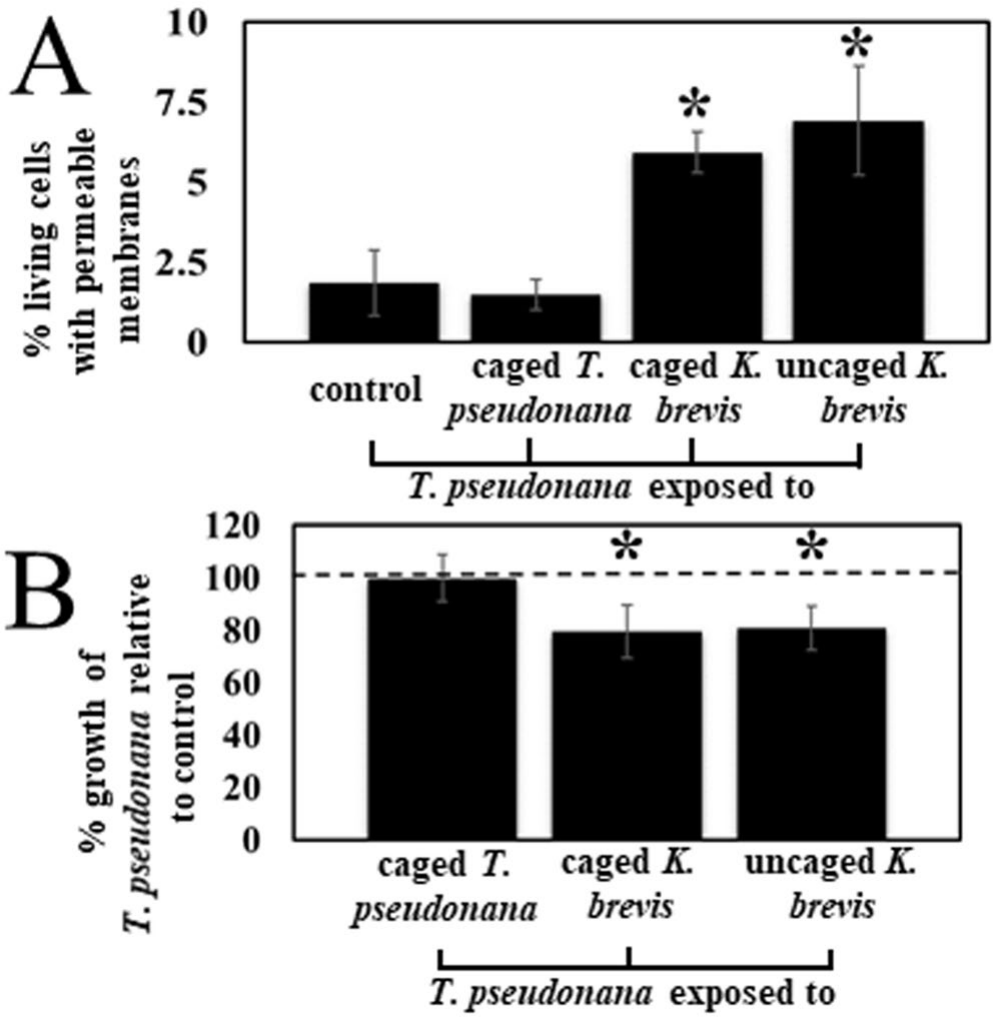
Exposure of *T*. *pseudonana* to *K*. *brevis* led to cell membrane damage. (**A**) *K*. *brevis* allelopathy significantly decreased *T*. *pseudonana* membrane integrity as indicated by permeability of live *T*. *pseudonana* cells measured by co-staining of SYTOX Green (stains nucleus of cells with permeable cell membranes) and Neutral Red (stains cytoplasm of live cells only via vacuole uptake; N = 5; asterisk denotes statistically significant difference between treatment and dilute media controls via Bonferroni-corrected t-test; p = 0.0164 for caged *K*. *brevis*, p = 0.0113 for uncaged *K*. *brevis; α* = 0.0167). (**B**) Presence of *K*. *brevis*, caged or uncaged, significantly decreased growth of *T*. *pseudonana* while exposure to caged *T*. *pseudonana* had no effect relative to dilute media controls (asterisk denotes statistical difference between control and treatment; p < 0.0001 for caged *K*. *brevis*, p = 0.0005 for uncaged *K*. *brevis*).

Upon inspection of the identities of disrupted lipids in the current study, two major classes of lipids common to cell and thylakoid membranes were noted as being significantly affected by allelopathy: SQDGs and MGDGs. Prince *et al*. had previously found cell membranes of some competing algae to be disrupted upon exposure to *K*. *brevis* allelopathy^27^; however, *T*. *pseudonana* was not tested in that study. In the current study, following exposure to *K*. *brevis* for six days, the proportion of living *T*. *pseudonana* cells with permeable and therefore damaged cell membranes was significantly greater than for unexposed *T*. *pseudonana* (Figs 3 and S2). *T*. *pseudonana* exposed to caged or uncaged *K*. *brevis* experienced similarly suppressed growth rates and increased membrane permeability relative to dilute media controls, suggesting that cell-cell physical contact does not compound the effect of allelopathy on membrane stability. In contrast to the negative effects of *K*. *brevis* exposure, the exposure of *T*. *pseudonana* to caged members of its own species was not significantly different than *T*. *pseudonana* exposed to cages containing dilute media. Overall, these findings confirm that one of the major mechanisms or outcomes of allelopathy is via cell membrane disruption, possibly due to a decreased concentration of membrane lipids such as SQDGs and MGDGs.

## Discussion

### Dominant impact of allelopathy on competitor lipidome

The major metabolic responses of two model algae indicate that a dominant consequence of *K*. *brevis* allelopathy is increased membrane permeability and decreased photosynthetic capability, both likely due to decreases in the concentrations of membrane- and thylakoid-associated lipids (Figs 1–3 and Table 1). Decreases in the concentrations of cell membrane associated PCs, SQDGs, and PGs (Tables 1 and S1) and permeabilization of the cell wall (Figs 3 and S2) suggested that allelopathy significantly disrupts membrane integrity by altering the lipid content without directly killing cells. Similarly, previously observed reduction in photosynthetic efficiency, as evidenced by decreases in the maximum quantum yield of photosystem II^27^ and in expression of 12 photosynthesis-related proteins^29^ could be due to damage to the thylakoid membrane and significant suppression of MGDGs, DGDGs, and SQDGs (Tables 1 and S1).

### *K*. *brevis* allelopathy damages competitor cell membranes

Allelopathy-induced cell membrane permeabilization and depolarization is common in terrestrial plants^36–38^; however, it does not appear to be well known in phytoplankton (but see ref.^27^). Fluorescent staining of *T*. *pseudonana* showed that *K*. *brevis* allelopathy significantly reduced cell membrane integrity in living *T*. *pseudonana* cells (Figs 3 and S2). This increased permeability could have been caused by decreased abundance of membrane-associated lipids (Table 1). Whether *T*. *pseudonana* and *K*. *brevis* cells were in physical contact did not affect competitive outcomes between the two species, indicating that allelopathy occurs via waterborne cues and is the dominant cause of reduced competitor growth and membrane destabilization (Fig. 3). This pattern of lipid disruption was also suggested by the polar metabolome of *T*. *pseudonana* when exposed to *K*. *brevis* allelopathy suggesting that one of the major outcomes of allelopathy prior to cell death is cell membrane destabilization^29^. Despite this prior report, no molecular mechanism of action for *K*. *brevis* allelopathy has been described to account for the observed phenotypic response. Copepod eggs were also found to have significantly altered membrane morphology after three days of feeding on *K*. *brevis*, suggesting that the allelopathic compounds released by *K*. *brevis* could possibly be responsible for more ubiquitous membrane damage in competitors and grazers^39^. The current work implicates decreases in PCs, SQDGs, and PGs as molecular underpinnings of cell membrane destabilization (Tables 1 and S1).

### Thylakoid-associated lipids are significantly disrupted by allelopathy

A second likely mechanism by which allelopathy affects competitor physiology is reduction of photosynthetic efficiency, which has been previously reported for diatom competitors exposed to *K*. *brevis*^27^. Previously, we showed that the concentrations of 12 photosynthesis-related proteins significantly decreased in response to *K*. *brevis* allelopathy^29^. Here we show that MGDGs, DGDGs, and SQDGs are also less abundant when competing algae are exposed to allelopathy (Tables 1, S1 and S2). MGDGs and DGDGs are the most abundant lipids in chloroplasts, constituting up to 80% of total plastidic lipid content^40^. In *Arabidopsis* leaves, higher MGDG content in the chloroplast led to maintenance of photosynthetic activity when subjected to ethylene-promoted senescence^41^. Additionally, plastids have been shown to elongate in mustard seedlings when exposed to high enough concentrations of the known allelochemical benzoic acid^42^. In general, the current findings suggest that a likely explanation for the observed loss in photosynthetic efficiency^27^ is destabilization of plastid membranes. While no direct experimental evidence exists in the current study that the number of plastids decreased or that thylakoid membranes became permeable, the downregulation of thylakoid-associated lipids and known consequences of allelopathy on plastids warrant additional tests of this hypothesis.

### Competitors display different responses to *K*. *brevis* allelopathy

Previous research using *K*. *brevis* culture filtrates and extra-cellular extracts showed that competing algae exhibit differential sensitivities to *K*. *brevis* allelopathy^24,25,27,29^. Although we cannot fully eliminate the possibility that resource limitation play a role in competition between *K*. *brevis* and diatoms given this particular co-culture design, our results demonstrate detrimental effects of competition on diatom physiology. Our previous work demonstrating that *K*. *brevis* produces a suite of allelopathic compounds^26^, coupled with the relatively nutrient rich environment of these co-cultures^29^, suggests that allelopathy is a major mechanism by which *K*. *brevis* competes. Herein we report that both competitor species studied, *A*. *glacialis* and *T*. *pseudonana*, are affected by allelopathy, although *T*. *pseudonana* was significantly more sensitive (Fig. 1, Tables 1, S1 and S2). These findings support the previously posited hypothesis that *A*. *glacialis* possesses resistance mechanism(s) to mitigate the physiological impacts of allelopathy^29^. The long-term geographic and seasonal co-occurrence of *A*. *glacialis* and *K*. *brevis* may have provided the selection pressure for *A*. *glacialis* to evolve resistance, which *T*. *pseudonana* has not yet acquired^29^.

Consideration of the specific classes of lipids most affected in *T*. *pseudonana* by allelopathy (PC, SQDG, PFAA, FFA, and taurolipids) suggests that a majority of lipids induced (PFAA, FFA, and SULF) by allelopathic exposure are either metabolic breakdown products or metabolic precursors of PCs and SQDGs, whose pools shrunk in *T*. *pseudonana* upon exposure to allelopathy. These findings allow for two possible mechanisms: the stress of allelopathic exposure leads to the degradation of complex lipids, such as SQDGs and PCs, to their less complex components; or, allelopathic compounds exuded by *K*. *brevis* inhibit the biosynthetic enzymes within *T*. *pseudonana* that are responsible for the anabolic synthesis of complex lipids from more simple building blocks, resulting in a build-up of biosynthetic precursors (PFAA, FFA) and a loss of more complex lipids (SQDG, PC). Breakdown of more complex lipids to smaller products has been observed in the green alga *Dunaliella salina*, where the degradation of MGDGs to DGDGs was observed using radiolabeled carbon precursors^43^. In the current case of *K*. *brevis* allelopathy affecting *T*. *pseudonana*, the observed increase in concentrations, rather than decrease, of lipid biosynthetic enzymes such as sulpholipid synthase and UDP-sulfoquinovose synthase^29^, favors the hypothesis that complex lipids are degraded in response to allelopathy.

Complementing the MS lipidomic analysis, NMR spectroscopic profiling revealed that additional essential metabolites in *T*. *pseudonana* were disrupted by *K*. *brevis* allelopathy. Aconitic acid, an intermediate in the TCA cycle, and malonic acid, intermediate building block for fatty acid synthesis, were both less abundant due to allelopathy, which could be explained by the degradation of these acids, an enhanced production of some fatty acids thus reducing cellular stocks of starting materials and intermediates, or the slowdown in anabolism of these acids^44,45^. Three methylated and acetylated amino acids were also suppressed which point to alterations in amino acid metabolism as a consequence of allelopathic exposure. None of the five compounds identified via NMR spectroscopic profiling were identified as components of the MS-based model, highlighting the complementary nature of NMR and MS profiling.

### *K*. *brevis’* sub-lethal allelopathy provides a unique study system

*K*. *brevis* is relatively mild among allelopathic algae, some of which release compounds that are acutely toxic to other algae^46–50^. *K*. *brevis* allelopathy acts slowly on competitors, affecting cell viability and population growth over days by weakening cell membranes, impeding photosynthesis, and disrupting osmoregulation^29^. In contrast, *Alexandrium* dinoflagellates can cause lysis of competitor cells in minutes to hours^46,50^. The freshwater dinoflagellate *Peridinium aciculiferum* produces bubbles that later burst leading to cell death in its competitor *Rhodomonas lacustris*^49^. Due to the slow acting allelopathic effects of *K*. *brevis*, we were able to observe the disruption of lipid metabolism, which ultimately leads to catastrophic effects on membrane integrity and photosynthesis in some competing algae (Figs 3 and S2, Table 1).

### Lipidomics provides essential knowledge on how competitors respond to allelopathy

In the current study focused on lipidomics, we show that the major effects of *K*. *brevis* allelopathy on *T*. *pseudonana* are permeabilization of cell membranes and significant decreases in the concentrations of membrane-associated lipids, including those typically associated with the thylakoid and cell membranes (Figs 3 and S2, Tables 1, S1 and S2). This complements previous findings regarding the polar metabolome and proteome in which we showed that *T*. *pseudonana* suffered significantly increased glycolysis activity, suppressed osmoregulation, enhanced enzymatic capabilities related to oxidative stress, as well as an increase in proteins associated with the pentose phosphate pathway and anti-trypsin protease inhibitors, when exposed to *K*. *brevis* allelopathy^29^. *K*. *brevis* allelopathy is, however, known to be highly variable and does not typically lead to devastating effects on cell growth^51^. The integration of metabolomics involving both polar and non-polar classes with proteomics analysis suggests that *K*. *brevis* allelopathy drastically affects *T*. *pseudonana* cellular functions on many levels. Cell membrane permeability, significantly altered metabolism, and decreased photosynthesis together paint a dreary picture for *T*. *pseudonana*. The current lipidomics work offers a glimpse into the robust metabolome of a more resistant competitor, *A*. *glacialis*, providing unique opportunities for exploring mechanisms of resistance to allelopathy.

## Materials and Methods

### Generation and preparation of extracts for lipidomics

Lipids were accessed from intracellular phytoplankton extracts obtained for the experiment described by Poulson-Ellestad *et al*.^29^. Briefly, diatoms *Thalassiosira pseudonana* strain CCMP 1335 and *Asterionellopsis glacialis* strain CCMP 137 were grown in silicate-amended L1 media in artificial seawater (Instant Ocean, 35 ppt). *Karenia brevis* strain CCMP 2228 was cultured in similar conditions above with L1 media-amended artificial seawater. All cultures were maintained at 21 °C with a 12:12 light/dark cycle and an irradiance of 100–145 µmol/m^2^ s in a Percival incubator (Biospherical Instrument QSL2100). Diatom cell concentrations were quantified via *in vivo* fluorescence as a proxy for cell concentration or by visual counts under microscope. A Fluid Imaging Technologies FlowCam was used to determine *K*. *brevis* cell concentrations.

To expose competitors to allelopathic *K*. *brevis*, *K*. *brevis* was co-cultured with each of the two diatom species (n = 14 per species). *K*. *brevis* was grown inside a permeable dialysis membrane (SpectraPor 7, MWCO 50 kDa) to allow for exchange of exuded allelopathic compounds without direct interaction of *K*. *brevis* and diatom cells, which were grown in flasks in which the dialysis tubes were placed. Control cultures consisted of dialysis membranes filled with L1 media diluted to conditions similar to that of exponential growth phase *K*. *brevis*^29^ (n = 15 per diatom species) in order to account for the potential of nutrient limitation on the lipidomes of competitors. This co-culture experiment was halted once competitor cultures reached exponential growth stage, which was 6 d for *T*. *pseudonana* and 8 d for *A*. *glacialis*, after which diatom cells were filtered onto GF/C filters (Whatman #1922–110, muffled at 450 °C for 3 h) and dipped into liquid nitrogen to quench intracellular metabolism. Filtered cells were stored at −80 °C until extraction. Cells were ground with a liquid nitrogen-cooled mortar and pestle, and extracted with 30 mL of an ice-cold mixture of 3:1:2 methanol/acetone/acetonitrile. Particulate matter was removed via centrifugation (5 min at 0 °C, 1,460xg) and the supernatant was removed. Cell pellets were rinsed twice with 10 mL of fresh solvent mixture and a final 3 mL rinse. Rinses were added to the supernatant and solvent was removed *in vacuo*.

To separate polar and lipid intracellular metabolites, dried extracts were dissolved in a biphasic mixture of 9:10:15 water/methanol/chloroform. The more lipophilic layer was removed and washed twice with 9:10 water/methanol. Polar metabolites of diatoms undergoing stress from competition with *K*. *brevis* were previously reported by Poulson-Ellestad *et al*.^29^. For the current work, the lipid-soluble chloroform-methanol fractions were analyzed by ^1^H NMR spectroscopy and ultrahigh performance liquid chromatography mass spectrometry (UHPLC/MS) metabolomics. (See *SI Materials and Methods* for sample preparation, procedures, and analytical method parameters).

### Metabolomics analysis

Prior to multivariate modeling, NMR^52^ and MS spectral data were preprocessed (See *SI Materials and Methods*). Principal component analysis (PCA) and orthogonal partial least squares discriminant analysis (oPLS-DA) models were generated to investigate the effect of *K*. *brevis* allelopathy on algal lipidomes (MATLAB and PLS Toolbox, version 7.9.1, Eigenvector research). Spectral features with discriminatory power in NMR-based models were annotated using the Human Metabolome Database and Chenomx Profiler while MS-based features were annotated using LOBSTAHS^53^, KEGG, LIPID MAPS, and Metlin databases^53–58^. Pooled extracts of each species were used to collect 2D NMR spectral data including: correlation spectroscopy (COSY), heteronuclear single quantum coherence (HSQC), and heteronuclear multiple bond correlation spectroscopy (HMBC) to aid in annotation.

### Membrane permeability assay

To detect differences in membrane permeability between *T*. *pseudonana* grown in the presence vs. absence of *K*. *brevis*, 100 mL media bottle containing 50 mL silicate-amended L1 media were inoculated with *T*. *pseudonana* to a concentration of ~1.0 × 10^4^ cells mL^−1^. Cages made of 50 mL falcon tubes with 1 μm mesh bottoms containing 25 mL of either silicate-amended L1 media diluted to similar conditions to that of exponential growth phase *K*. *brevis*^29^ (control, n = 5), *K*. *brevis* (~1.0 × 10^4^ cells mL^−1^) (treatment, n = 5), or *T*. *pseudonana* (~1.0 × 10^4^ cells mL^−1^) (treatment, n = 5) were added to the *T*. *pseudonana* cultures. In a fourth treatment 25 mL of *K*. *brevis* (~1.0 × 10^4^ cells mL^−1^) were added directly to the *T*. *pseudonana* cultures without a cage (n = 5) but with the same total volume as for the other treatments. Cultures were rearranged daily and cages were gently moved up and down twice per day to maximize exposure of *T*. *pseudonana* to the allelopathic compounds of *K*. *brevis*. After five days, two 500 μL samples of *T*. *pseudonana* were removed from experimental flasks. The first sample was preserved with an acidified Lugol’s solution for measurement of total cell concentration. The second sample was stained by direct addition of 20 μL Neutral Red solution (0.05% w:v in deionized water, Mallinckrodt Chemical) followed by the addition of 10 μL SYTOX Green solution (50 μM in DMSO, Invitrogen Molecular Probes, detected with excitation at 504 nm and emission at 523 nm) 30 min later, to test for live and permeable cells, respectively. The stained cells were incubated in darkness at room temperature for 1 hr prior to counting to allow *T*. *pseudonana* to uptake dyes. All cells were counted within 6 hrs of staining, using fluorescence and bright-field microscopy on an Olympus IX-50 inverted microscope with a Palmer-Maloney settling chamber, at 400X magnification (40X objective and 10X eyepiece). This enabled determination of the ratio of living cells with permeable membranes (red and fluorescent green) to the total number of living cells (red but not fluorescent green). Bonferroni corrected unpaired t-tests (α = 0.0167 for three comparisons) were conducted to test statistical significance of membrane destabilization and growth rate suppression of *T*. *pseudonana* exposed to dilute media controls vs. the other three treatments using Prism graphpad version 4.0. The membrane permeability assay was completed on three separate occasions with similar outcomes.

### Data availability

Data generated during and/or analyzed during the current study are available in the BMO-DCO repository, www.bco-dmo.org/project/528925.

## Electronic supplementary material

Supplementary Information
