# Supplementary material for: Karenia brevis allelopathy compromises the lipidome, membrane integrity, and photosynthesis of competitors
Source: Sci Rep. 2018 Jun 22;8:9572. doi: 10.1038/s41598-018-27845-9 (PMC6015087; doi:10.1038/s41598-018-27845-9)
Supplement: Supplementary file 1 — Supplementary Information [file 41598_2018_27845_MOESM1_ESM.pdf]

Karenia brevis allelopathy compromises the lipidome, membrane integrity, and photosynthesis of competitors

Remington X. Poulin[1,4], Scott Hogan[1], Kelsey L. Poulson-Ellestad[2,3], Emily Brown[2,4], Facundo M. Fernández[1,4,5], Julia Kubanek[1,2,4,5*].

[1]School of Chemistry and Biochemistry, Georgia Institute of Technology, 901 Atlantic Dr, Atlanta, GA, 30332, USA
[2]School of Biological Sciences, Georgia Institute of Technology, 950 Atlantic Dr, Atlanta, GA, 30332, USA
[3]Department of Biological, Chemical, and Physical Sciences, Roosevelt University, 430 S Michigan Avenue, Chicago, IL 60605, USA
[4]Aquatic Chemical Ecology Center, Georgia Institute of Technology, Atlanta, GA 30332, USA
[5]Parker H. Petit Institute of Bioengineering and Bioscience, Georgia Institute of Technology, Atlanta, GA 30332, USA
*julia.kubanek@biosci.gatech.edu

SI Materials and Methods

NMR sample preparation and data acquisition. To compare equivalent metabolomes, each lipid extract was prepared from a total of 1.52 x 10^6 *A. glacialis* cells or 2.56 x 10^6 *T. pseudonana* cells. ^{1}H NMR spectra were collected for all samples on a Bruker Avance 500 MHz AVIIIHD NMR spectrometer equipped with a 5 mm broadband direct detection probe using an excitation-sculpting gradient pulse.[1] Extracts were reconstituted in 250 µL d_6-DMSO (99.9% atom d_6-DMSO; Cambridge Isotope Labs) containing 0.1% trimethylsilane (TMS) as an internal standard in 3 mm NMR tubes. Spectra of each extract were compiled from 256 scans.

Spectra were preprocessed in NMRLab version 3.5.0.0 [2] in MATLAB R2013a version 8.1.0.604. TMS was used to align spectra at 0.00 ppm. All spectra were manually phased and baseline corrected prior to the spectral regions around TMS (-2.00 to 0.50), DMSO (2.35 to 2.70), water (3.30 to 3.50), and unoccupied downfield region (7.75 to 8.50) being removed in spectra of *A. glacialis* lipid extracts. The spectral regions around TMS (-2.00 to 0.50), DMSO (2.45 to 2.57), water (3.14 to 3.19), a contaminant peak (4.00 to 4.20), and unoccupied downfield region (7.75 to 8.50) were removed in spectra of *T. pseudonana* lipid extracts. All spectra were binned (0.005 ppm), probabilistic quotient normalized[3] to correct for minute differential dilution among samples, and generalized log (glog) transformed to reduce bias toward higher concentration metabolites. A single batch of culture of each diatom species was used to generate a set of five quality control extracts using the above methods. Quality control samples were used to obtain glog optimized lambda values of 1.3093 x 10^{-8} for *A. glacialis* extracts and 8.4010 x 10^{-9} for *T. pseudonana* extracts.

UHPLC/MS DATA COLLECTION: Lipid extracts were reconstituted in 200 µL 2-propanol. Quantitative metabolomics data were acquired using a Waters Xevo G2 QTOF mass

spectrometer. The instrument was operated in negative electrospray ionization mode with a capillary voltage of -2.0 kV and a sampling cone voltage of 30 V. The source temperature of 90 °C was maintained throughout the experiment. Nitrogen was used as a desolvation gas at 250 °C with a flow rate of 600 L/h. The mass spectrometer was calibrated across the 50-1200 Da mass range using a sodium formate solution. Leucine Enkephalin was infused at a flow rate of 2 μL/min and acquired as a lockmass correction. Run order was randomized and samples were acquired in duplicate. Pooled quality control samples were acquired after every twelfth sample injection to monitor instrumental drift and minimize batch effects.

Chromatographic separation was accomplished using a Waters Acquity UPLC quaternary solvent manager system fitted with a Waters ACQUITY UPLC BEH C18 column (1.7-μm particle size, 2.1 × 50 mm), with an injection volume of 10 μL. The column was operated at 60 °C, while the autosampler tray was maintained at 5 °C. Mobile phase A contained water: acetonitrile (40:60) and mobile phase B contained 10% acetonitrile in 2-propanol. A flow rate of 300 μL/min was used with the following gradient: 0-1 min, 70% B; 1-3 min, 75% B; 3-6 min, 80% B; 6-10 min, 90% B; 10-14 min, 100% B. Both mobile phases included 10 mM ammonium formate (Sigma Aldrich, >99.995%) and 0.1% formic acid (Fluka Analytical) additives to improve peak shape and ionization efficiency. All solvents used were of LCMS grade and provided by OmniSolv (water, acetonitrile) or Honeywell (2-propanol).

UHPLC/MS Data Processing. Data were imported into Progenesis QI for chromatographic alignment, de-isotoping, adduct deconvolution, normalization, and peak picking. Peaks detected in the sample blanks at greater than 10% of the average sample intensity were removed as potential contaminants. The corresponding normalized intensities across each sample for every feature (m/z, retention time pair) were imported into Matlab for multivariate analysis.

MS Metabolomics Statistical Analyses. PCA and oPLS-DA plots were constructed using PLS toolbox version 8.1 in Matlab. PCA plots are provided for both cell types to assess PCA scores of treatment vs. controls. PCA is an unsupervised analysis technique that reduces dimensionality in the data in order to visualize multivariate matrices in a linear space. It can be helpful in observing clustering patterns in the data without potential overfitting imposed by imparting class information.

For oPLSDA, plots were orthogonalized such that the maximum variance between classes is produced across the first latent variable (LV), with all other LVs explaining within class variance. Data were autoscaled and the model containing the fewest LVs that produced the lowest cross-validated error was selected. Venetian blinds cross validation was employed with six data splits for analysis of treatment/control effects of each cell type, while eight data splits were used when comparing all samples (n=61) including pooled quality controls. Significant peaks, defined as $p<0.05/n$ (number of features, n = 322 for *A. glacialis*, n = 360 for *T. pseudonana*) were identified using a 2-tailed t-test with unequal variance following Bonferroni correction for multiple comparisons. To normalize the significance cutoff for both competitor species at $\alpha = 0.05$ (Figure 2), p-values were adjusted by multiplying p-values by n instead of dividing α by n.

Metabolite Annotation. Tandem MS experiments were performed on Thermo Q-Exactive HF quadrupole-Orbitrap mass spectrometer using the top 10 Data Dependent Analysis (DDA) method to select and fragment all ions of interest with resolution=30,000, automatic gain control (AGC) = 1e5, max injection time (IT) = 30 ms, and a stepped normalized collision energy (NCE) ranging from 10 to 50.

All features with significant differences between control and treatment groups for either cell type were analyzed by MS/MS to elucidate structure. Following adduct analysis, elemental formulae were determined based on exact mass and isotopic distribution. Features with exact masses corresponding to matches in the LOBSTAHS database were tentatively identified, with identities confirmed by matching headgroup fragments and fatty acid chains from the MS/MS spectra to tentative identities.[4] For those features without LOBSTAHS matches, identifications were performed by hand using known lipid fragmentation patterns and cross-checked against other the KEGG, Metlin and LIPID MAPS databases.[5-7]

SI Figures/Tables

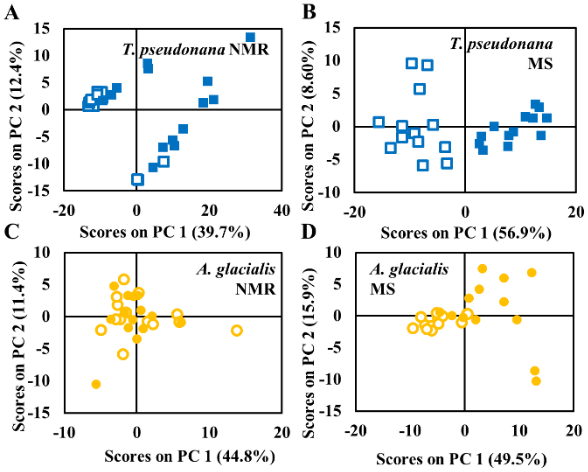

Supplementary Figure 1: PCA models fail to fully differentiate between lipidomes of *Thalassiosira pseudonana* and *Asterionellopsis glacialis* exposed or not exposed to *Karenia brevis* allelopathy. Filled symbols represent lipidomes of algae exposed to *K. brevis* through molecule-permeable but cell impermeable membranes, empty symbols represent lipidomes from unexposed algae (controls). PCA model generated from (*A*) ^{1}H NMR spectral data and (*B*) from UHPLC/MS metabolic features from lipid-soluble extracts of *T. pseudonana* (blue squares variance captured along each principal component is stated in parentheses). PCA model generated from (*C*) ^{1}H NMR spectral data and (*D*) from UHPLC/MS metabolic features from lipid-soluble extracts of *A. glacialis* (yellow circles).

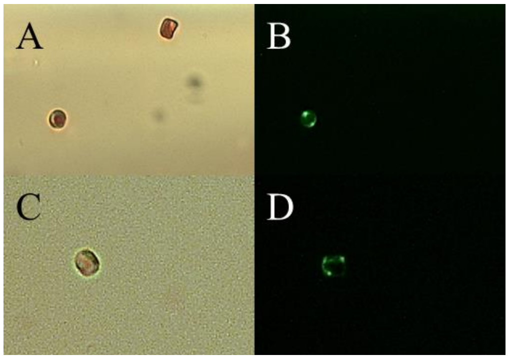

Supplementary Figure 2: *K. brevis* allelopathy damages *T. pseudonana* cell membranes. (*A and C*) Brightfield imaging of *T. pseudonana* exposed to caged *K. brevis* and stained with Neutral Red (indicates live cells). (*B and D*) Fluorescence imaging of *T. pseudonana* exposed to caged *K. brevis* and stained with SYTOX Green (indicates permeable cell membranes). Simultaneous red and fluorescent green staining signify living *T. pseudonana* with permeable, damaged cell membranes.

Supplementary Table 1: Identification of metabolites via MS metabolomics analysis whose concentrations were significantly different when *T. pseudonana* was exposed to *K. brevis* vs. controls. Observed *m/z* and parts-per-million mass error (PPM), adduct, elemental formula, and molecular composition (full fatty acid chain information) are provided where possible. Fold change values are shown as positive when relative abundance of metabolite increased when *T. pseudonana* was exposed to *K. brevis* allelopathy and negative when abundances decreased. Annotation confidence ranged from 1-3. Confidence level 1: observed MS/MS data consistent with predicted spectrum and LOBSTAHS exact mass match to corresponding lipid class; 2: observed MS/MS data consistent with predicted spectrum; 3: observed exact mass match to LOBSTAHS database and/or partial MS/MS structural determination.[4]
* Fold change value uncertain due to extremely low concentration of metabolites in control samples

m/z **error (PPM)**	**Adduct**	**Elemental formula**	**Identity**	**Fold change**	**p value**	**Conf.**	**Lipid Class**
**563.3986 0.71**	$[M-H]^-$	$C_{30}H_{60}O_7S$	Myristoyl sulfohydroxypalmitic acid	>25*	2.39E-05	2	Other (sulfur containing)
**577.4146 1.39**	$[M-H]^-$	$C_{31}H_{62}O_7S$	Myristoyl sulfohydroxyheptadecanoic acid	>25*	3.83E-06	2	Other (sulfur containing)
**591.4308 2.20**	$[M-H]^-$	$C_{32}H_{64}SO_7$	Pentadecanoyl sulfohydroxyheptadecanoic acid	>25*	1.88E-06	2	Other (sulfur containing)
**379.2148 7.12**	$[M-H]^-$	$C_{21}H_{32}O_6$	FFA(21:5 + 4 O)	>25*	4.25E-5	3	Free fatty acid
**607.4243 1.81**	$[M-H]^-$	$C_{32}H_{64}O_8S$	Pentadecanoyl sulfohydroxyheptadecanoic acid	>25*	2.77E-5	2	Other (sulfur containing)
**619.4249 0.81**	$[M-H]^-$	$C_{33}H_{64}SO_8$	Hydroxypentadecanoyl sulfooleic acid	>25*	1.96E-05	2	Other (sulfur containing)
**572.4354 0.87**	$[M-H]^-$	$C_{32}H_{63}NO_5S$	Pentadecanoyl sulfate heptadecenamide	>25*	5.77E-07	2	Other (sulfur containing)
**367.2127 1.63**	$[M-H]^-$	$C_{20}H_{32}O_6$	FFA(20:4 + 4 O)	>25*	2.75E-05	3	Free fatty acid
**575.4356 1.91**	$[M-H]^-$	$C_{32}H_{64}O_6S$	Pentadecanoyl sulfoheptadecanoic acid	>25*	4.38E-07	2	Other (sulfur containing)
**592.4942 0.17**	$[M-H]^-$	$C_{36}H_{67}NO_5$	*N*-heptadecenoic acid oleamide	>25*	3.82E-06	2	Fatty acid amide
**665.5117 3.31**	$[M-H]^-$	$C_{35}H_{74}N_2O_7S$	Hexadecenoyl *N*-sulfanediol dihydroxynonadecane-diamide	>25*	3.04E-06	2	Other (sulfur containing)
**257.2125 3.11**	$[M-H]^-$	$C_{15}H_{30}O_3$	FFA(15:0 + 1 O)	>25*	7.05E-05	3	Free fatty acid
**616.4615 0.65**	$[M-H]^-$	$C_{34}H_{67}NO_6S$	*N*-sulfo,*N*-pentadecanoyl nonadecanamide	>25*	1E-08	2	Other (sulfur containing)
**561.4194 0.89**	$[M-H]^-$	$C_{31}H_{62}O_6S$	Myristoyl sulfoheptadecanoic acid	>25*	1.9E-06	2	Other (sulfur containing)

**787.5583 0.76**	$[M+HCOO]^-$	$C_{42}H_{78}O_{10}$	MGDG(33:1)	20.	8.84E-06	3	Monogalactosyl diacylglycerol
**574.4516 1.91**	$[M-H]^-$	$C_{32}H_{65}NO_5S$	Pentadecanoyl sulfate heptadecanamide	14	9.71E-08	2	Other (sulfur containing)
**578.4790 1.04**	$[M-H]^-$	$C_{35}H_{65}NO_5$	*N*-palmitoleic acid oleamide	8.5	2.14E-06	2	Fatty acid amide
**564.4639 1.95**	$[M-H]^-$	$C_{34}H_{63}NO_5$	*N*-pentadecenoic acid oleamide	7.1	7.94E-07	2	Fatty acid amide
**566.4792 1.41**	$[M-H]^-$	$C_{34}H_{65}NO_5$	*N*-pentadecanoic acid oleamide	7.0	6.79E-08	2	Fatty acid amide
**686.4778 1.75**	$[M-H]^-$	$C_{37}H_{70}NO_8P$	PE(16:1_16:1)	6.5	3.54E-05	1	Phosphatidylethanolamine
**550.4480 1.64**	$[M-H]^-$	$C_{33}H_{61}NO_5$	*N*-pentadecenoic acid heptadecanamide	5.8	9.45E-06	2	Fatty acid amide
**682.5090 1.47**	$[M-H]^-$	$C_{39}H_{73}NO_6S$	*N*-sulfo,*N*-octadecanoyl heneicosanamide	5.5	4.23E-05	2	Other (sulfur containing)
**651.4958 3.68**	$[M-H]^-$	$C_{34}H_{72}N_2O_7S$	Pentadecenoyl *N*-sulfanediol dihydroxynonadecane-diamide	3.3	3.48E-04	2	Other (sulfur containing)
**793.5113 3.53**	$[M-H]^-$	$C_{41}H_{78}O_{12}S$	SQDG(16:0_16:0)	-2.0	5.05E-05	1	Sulfoquinovosyldiacylglycerol
**763.4646 1.83**	$[M+HCOO]^-$	$C_{41}H_{66}O_{10}$	MGDG(16:3_16:3)	-2.1	4.05E-05	1	Monogalactosyl diacylglycerol
**791.4988 0.38**	$[M-H]^-$	$C_{41}H_{76}O_{12}S$	SQDG(16:0_16:1)	-2.2	4.52E-05	1	Sulfoquinovosyldiacylglycerol
**771.5272 1.04**	$[M+HCOO]^-$	$C_{41}H_{74}O_{10}$	MGDG(16:1_16:1)	-2.5	2.6E-06	1	Monogalactosyl diacylglycerol
**719.4879 1.39**	$[M-H]^-$	$C_{38}H_{73}O_{10}P$	PG(16:0_16:1)	-2.6	4.89E-07	1	Phosphatidylglycerol
**737.4527 1.63**	$[M-H]^-$	$C_{37}H_{70}O_{12}S$	SQDG(14:0_14:0)	-2.6	7.99E-08	1	Sulfoquinovosyldiacylglycerol
**779.4988 0.38**	$[M-H]^-$	$C_{40}H_{76}O_{12}S$	SQDG(15:0_16:0)	-2.6	7.16E-06	1	Sulfoquinovosyldiacylglycerol
**813.4798 3.07**	$[M-H]^-$	$C_{43}H_{74}O_{12}S$	SQDG(16:0_18:4)	-2.7	1.79E-06	1	Sulfoquinovosyldiacylglycerol
**765.4723 1.44**	$[M-H]^-$	$C_{42}H_{71}O_{10}P$	PG(16:1_20:5)	-2.8	2.33E-02	1	Phosphatidylglycerol
**769.5111 0.39**	$[M+HCOO]^-$	$C_{41}H_{72}O_{10}$	MGDG(16:0_16:3)	-2.8	1.30E-03	1	Monogalactosyl diacylglycerol
**977.5477 0.20**	$[M+HCOO]^-$	$C_{51}H_{80}O_{15}$	DGDG(16:3_20:5)	-2.8	1.07E-02	1	Digalactosyldiacylglycerol
**759.4368 1.19**	$[M-H]^-$	$C_{39}H_{68}O_{12}S$	SQDG(14:0_16:3)	-2.9	1.42E-02	1	Sulfoquinovosyldiacylglycerol
**751.4681 1.20**	$[M-H]^-$	$C_{38}H_{72}O_{12}S$	SQDG(14:0_15:0)	-3.1	2.93E-08	1	Sulfoquinovosyldiacylglycerol

**765.4796 3.53**	[M-H]-	$C_{39}H_{74}O_{12}S$	SQDG(14:0_16:0)	-3.2	1.69E-08	1	Sulfoquinovosyldiacylglycerol
**691.4565 1.30**	[M-H]-	$C_{36}H_{69}O_{10}P$	PG(14:0_16:1)	-3.2	9.85E-07	1	Phosphatidylglycerol
**819.5268 0.49**	[M+HCOO]-	$C_{45}H_{74}O_{10}$	MGDG(16:1_20:5)	-3.3	9.7E-05	1	Monogalactosyldiacylglycerol
**791.4989 0.51**	[M-H]-	$C_{41}H_{76}O_{12}S$	SQDG(14:0_18:1)	-2.2	4.52E-05	1	Sulfoquinovosyldiacylglycerol
**745.5114 1.61**	[M+HCOO]-	$C_{39}H_{72}O_{10}$	MGDG(14:0_16:1)	-3.6	2.05E-06	1	Monogalactosyldiacylglycerol
**748.5145 2.14**	[M+HCOO]-	$C_{38}H_{74}NO_8P$	PC(14:0_16:1)	-3.7	2.59E-06	1	Phosphatidylcholine
**761.4489 3.41**	[M-H]-	$C_{39}H_{70}O_{12}S$	SQDG(14:0_16:2)	-3.9	9.06E-06	1	Sulfoquinovosyldiacylglycerol
**799.4681 1.13**	[M-H]-	$C_{42}H_{72}O_{12}S$	SQDG(15:0_18:4)	-3.9	7.44E-05	1	Sulfoquinovosyldiacylglycerol
**743.4965 2.69**	[M+HCOO]-	$C_{39}H_{70}O_{10}$	MGDG(14:0_16:2)	-4.0	1.75E-05	1	Monogalactosydiacylglycerol
**802.5609 1.37**	[M+HCOO]-	$C_{42}H_{80}NO_8P$	PC(16:1_18:1) / PC(16:0_18:2)	-4.0	9.41E-06	1	Phosphatidylcholine
**907.5639 0.33**	[M+HCOO]-	$C_{45}H_{82}O_{15}$	DGDG(14:0_16:1)	-4.0	4.9E-06	1	Digalactosyldiacylglycerol
**762.5017 35.1**	[M+HCOO]-	$C_{39}H_{76}NO_8P$	PC(15:0_16:1)	-4.0	8.47E-06	1	Phosphatidylcholine
**822.5293 0.24**	[M+HCOO]-	$C_{44}H_{76}NO_8P$	PC(16:1_20:5)	-4.1	4.57E-08	1	Phosphatidylcholine
**841.5108 3.92**	[M-H]-	$C_{45}H_{78}O_{12}S$	SQDG(36:4)	-4.2	1.24E-05	3	Sulfoquinovosyldiacylglycerol
**935.5961 1.28**	[M+HCOO]-	$C_{47}H_{86}O_{15}$	DGDG(16:0_16:1)	-4.2	1.8E-07	1	Digalactosyldiacylglycerol
**757.4210 1.06**	[M-H]-	$C_{39}H_{66}O_{12}S$	SQDG(14:0_16:4)	-4.4	5.19E-06	1	Sulfoquinovosyldiacylglycerol
**820.5129 0.61**	[M+HCOO]-	$C_{44}H_{74}NO_8P$	PC(16:2_20:5)	-4.4	3.36E-06	1	Phosphatidylcholine
**583.3129 1.89**	[M-H]-	$C_{30}H_{48}O_{11}$	MGDG(21:4+1 O)	-4.5	1.23E-06	3	Monogalactosyldiacylglycerol
**773.5427 0.78**	[M+HCOO]-	$C_{41}H_{76}O_{10}$	MGDG(16:0_16:1)	-4.5	8.48E-07	1	Monogalactosyldiacylglycerol
**795.5265 0.13**	[M+HCOO]-	$C_{43}H_{74}O_{10}$	MGDG(16:0_18:4)	-4.5	6.45E-06	1	Monogalactosyldiacylglycerol
**829.4763 1.09**	[M-H]-	$C_{43}H_{74}O_{13}S$	SQDG(34:4 + 1O)	-4.8	0.000138	3	Sulfoquinovosyldiacylglycerol
**818.4976 0.24**	[M+HCOO]-	$C_{44}H_{72}NO_8P$	PC(16:3_20:5) / PC(18:4_18:4)	-5.1	2.69E-07	1	Phosphatidylcholine
**850.5604 0.00**	[M+HCOO]-	$C_{46}H_{80}NO_8P$	PC(18:1_20:5)	-5.3	4.66E-06	1	Phosphatidylcholine
**870.5292 0.11**	[M+HCOO]-	$C_{48}H_{76}NO_8P$	PC(20:5_20:5)	-5.3	6.87E-07	1	Phosphatidylcholine
**796.5138 1.13**	[M+HCOO]-	$C_{42}H_{74}NO_8P$	PC(14:0_20:5) / PC(16:1_18:4)	-6.3	3.49E-07	1	Phosphatidylcholine
**824.5454 0.85**	[M+HCOO]-	$C_{44}H_{78}NO_8P$	PC(16:0_20:5)	-6.4	1.25E-08	1	Phosphatidylcholine

**896.5448 0.11**	$[M+HCOO]^-$	$C_{50}H_{78}NO_8P$	PC(20:5_22:6)	-6.7	3.46E-07	1	Phosphatidylcholine
**800.5449 0.25**	$[M+HCOO]^-$	$C_{42}H_{78}NO_8P$	PC(16:0_18:3)	-6.9	4.77E-05	1	Phosphatidylcholine
**798.5286 0.63**	$[M+HCOO]^-$	$C_{42}H_{76}NO_8P$	PC(16:0_18:4)	-7.8	5.7E-05	1	Phosphatidylcholine
**844.5135 0.12**	$[M+HCOO]^-$	$C_{46}H_{74}NO_8P$	PC(18:4_20:5)	-8.5	1.41E-05	1	Phosphatidylcholine
**848.5448 0.12**	$[M+HCOO]^-$	$C_{46}H_{78}NO_8P$	PC(18:2_20:5) / PC(16:1_22:6)	-8.5	5.3E-06	1	Phosphatidylcholine
**821.5427 1.46**	$[M+HCOO]^-$	$C_{45}H_{76}O_{10}$	MGDG(36:5)	-9.3	2.82E-06	3	Monogalactosyl diacylglycerol
**843.5266 0.24**	$[M+HCOO]^-$	$C_{47}H_{74}O_{10}$	MGDG(18:3_20:5)	-11	6E-05	1	Monogalactosyl diacylglycerol

Supplementary Table 2: Identification of metabolites via MS metabolomics analysis whose concentrations were significantly different when *A. glacialis* was exposed to *K. brevis* vs. controls. Observed *m/z* and parts-per-million mass error (PPM), adduct, elemental formula, and molecular composition (full fatty acid chain information) are provided where possible. Fold change values are shown as positive when relative abundance of metabolite increased when *A. glacialis* was exposed to *K. brevis* allelopathy and negative when abundances decreased. Annotation confidence ranged from 1-3. Confidence level 1: observed MS/MS data consistent with predicted spectrum and LOBSTAHS exact mass match to corresponding lipid class; 2: observed MS/MS data consistent with predicted spectrum; 3: observed exact mass match to LOBSTAHS database and/or partial MS/MS structural determination.[4]

m/z **error (PPM)**	**Adduct**	**Elemental formula**	**Identity**	**Fold change**	**p value**	**Conf.**	**Lipid Class**
**591.4308 2.37**	$[M-H]^-$	$C_{32}H_{64}SO_7$	Pentadecanoyl sulfohydroxyheptadecanoic acid	4.9	4.24E-06	2	Other (sulfur containing)
**607.4243 1.81**	$[M-H]^-$	$C_{32}H_{64}O_8S$	Pentadecanoyl sulfohydroxyheptadecanoic acid	4.9	1.66E-05	2	Other (sulfur containing)
**616.4615 0.65**	$[M-H]^-$	$C_{34}H_{67}NO_6S$	*N*-sulfo,*N*-pentadecanoyl nonadecanamide	13.8	5.55E-05	2	Other (sulfur containing)
**578.4790 1.04**	$[M-H]^-$	$C_{35}H_{65}NO_5$	*N*-palmitoleic acid oleamide	12.2	7.2E-06	2	Fatty acid amide
**651.4958 3.68**	$[M-H]^-$	$C_{34}H_{72}N_2O_7S$	Pentadecenoyl *N*-sulfanediol dihydroxynonadecane-diamide	12.5	1.42E-05	2	Other (sulfur containing)
**653.5117 3.37**	$[M-H]^-$	$C_{34}H_{74}N_2O_7S$	Pentadecanoyl *N*-sulfanediol dihydroxynonadecane-diamide	16.4	4.57E-05	2	Other (sulfur containing)

References:

1 Poulson-Ellestad, K. L. *et al.* Metabolomics and proteomics reveal impacts of chemically mediated competition on marine plankton. *Proc Nat Acad Sci USA* **111**, 9009-9014, (2014).

2 Günther, U. L., Ludwig, C. & Ruterjans, H. NMRLAB-Advanced NMR data processing in matlab. *J Magn Reson* **145**, 201-208, (2000).

3 Dieterle, F., Ross, A., Schlotterbeck, G. & Senn, H. Probabilistic quotient normalization as robust method to account for dilution of complex biological mixtures. Application in 1H NMR metabonomics. *Anal Chem* **78**, 4281-4290, (2006).

4 Collins, J. R., Edwards, B. R., Fredricks, H. F. & Van Mooy, B. A. LOBSTAHS: An adduct-based lipidomics strategy for discovery and identification of oxidative stress biomarkers. *Anal Chem* **88**, 7154-7162 (2016).

5 Smith, C. A. *et al.* METLIN: a metabolite mass spectral database. *Ther Drug Monit* **27**, 747-751 (2005).

6 Kanehisa, M. The KEGG database. '*In Silico' Simulation of Biological Processes* **247**, 91-103 (2002).

7 Fahy, E. *et al.* Update of the LIPID MAPS comprehensive classification system for lipids. *J Lipid Res* **50**, S9-S14 (2009).
